# EMT, CTCs and CSCs in tumor relapse and drug-resistance

**DOI:** 10.18632/oncotarget.4037

**Published:** 2015-05-08

**Authors:** Abhisek Mitra, Lopa Mishra, Shulin Li

**Affiliations:** ^1^ Department of Pediatrics, The University of Texas MD Anderson Cancer Center, Houston, TX, USA; ^2^ Department of Gastroenterology, Hepatology and Nutrition, The University of Texas MD Anderson Cancer Center, Houston, TX, USA

**Keywords:** tumor relapse, CTCs, CSCs, EMT, clinical trials

## Abstract

Tumor relapse and metastasis are the primary causes of poor survival rates in patients with advanced cancer despite successful resection or chemotherapeutic treatment. A primary cause of relapse and metastasis is the persistence of cancer stem cells (CSCs), which are highly resistant to chemotherapy. Although highly efficacious drugs suppressing several subpopulations of CSCs in various tissue-specific cancers are available, recurrence is still common in patients. To find more suitable therapy for relapse, the mechanisms underlying metastasis and drug-resistance associated with relapse-initiating CSCs need to be identified. Recent studies in circulating tumor cells (CTCs) of some cancer patients manifest phenotypes of both CSCs and epithelial-mesenchymal transition (EMT). These patients are unresponsive to standard chemotherapies and have low progression free survival, suggesting that EMT-positive CTCs are related to co-occur with or transform into relapse-initiating CSCs. Furthermore, EMT programming in cancer cells enables in the remodeling of extracellular matrix to break the dormancy of relapse-initiating CSCs. In this review, we extensively discuss the association of the EMT program with CTCs and CSCs to characterize a subpopulation of patients prone to relapses. Identifying the mechanisms by which EMT-transformed CTCs and CSCs initiate relapse could facilitate the development of new or enhanced personalized therapeutic regimens.

## INTRODUCTION

Despite initially successful multimodal therapy that includes resection, chemotherapy and for some cases radiation therapy, tumor recurrence remains a major etiology of the morbidity and mortality in cancer patients. A systematic review of acquired relapse in cancer patients suggested that tumor cells undergo dynamic clonal evolution under the strong selective pressure of chemotherapy, radiation therapy and any other therapeutic intervention [1, 2]. These treatment-resistant clones of neoplastic cells show somatic mutations and phenotypic variations not present in their state of origin. Over the past decade, these subpopulations have been isolated using novel surface markers of CSCs to dissect the causes of inter- and intra- tumors heterogeneity [3]. The source of this subset of treatment-resistant, relapse-initiating and the dynamic evolution of these clones must be understood.

The location of a tumor recurrence relative to the primary tumor (local, regional or distant) is influenced predominantly by microenvironmental factors that provide an adaptive landscape for relapsed tumor cells. The adhesion of tumor cells to the extracellular matrix (ECM) drives the activation of certain signature genes that promote cancer progression or tissue-dependent dormancy [4, 5]. Thus, identifying these genetic alterations could reveal new avenues for preventing or treating tumor relapse and could improve the long-term survival of patients. As shown in Table 1, survival rate is associated with tumor recurrence in various types of cancer.

**Table 1:** Tissue-specific tumor recurrence rates and 5-year survival rates in patients with cancer

Tumor sites	Recurrence rate	5 years survival rate	References
Bladder	~40-70%	~15-98%	[86, 87]
Bone	~50%	~60-80%	[88]
Breast	~15-20%	~90-20%	[89, 90]
Brain	~85%	~10%	[91]
Colon	~18-32%	~6-74%	[92, 93]
Head and Neck	~24-33%	~50-66%	[94-96]
Kidney	~20-40%	~8-81%	[97]
Liver	~70%	~25-50%	[98, 99]
Lung	~10-24%	~40-60%	[100-102]
Ovary	~20-50%	~18-89%	[103, 104]
Pancreatic	~80%	~5-14%	[105]
Prostate	~30-44%	~99-100%	[106]
Testis	~4-14%	~74-99%	[107, 108]
Thyroid	~5-10%	~51-100%	[109, 110]
Uterus	~14-25%	~17-95%	[111-113]

A major cause of tumor relapse is an increasing number of CTCs and their downstream transformation into CSCs which initiate recurrence [6-9]. Notably, cases demonstrating chemo- or radio-resistance have high numbers of EMT transformed CTCs [10, 11]. Evidence from clinical studies suggests that poor survival of cancer patients has been linked with EMT phenotypes in malignant cancer cells [12-14].

Accumulating evidence shows that a subset of CTCs and CSCs have an EMT phenotype [15-19]. Another notable finding demonstrates that a subclone of CTCs can be induced to express phenotypes of CSCs [18, 20-23]. These discoveries suggest that EMT links CTCs and CSCs, enabling these cells to survive in the peripheral circulation and actively causing relapse. A better understanding of the etiology of the reprogramming switches that determine the progression through EMT, CTCs, dormancy and CSCs could pave the way towards clinically relevant drug targets.

In this review, we revisit the concept of relapse to introduce the notion of EMT transformed CTCs and CSCs. We highlight the most recent studies demonstrating the potential contributions of EMT positive CTCs and CSCs to recurrence and recommend a redesign of the therapeutic research on CSCs. Improving our understanding of these cells may help to categorize potential targets for novel therapies to preclude relapse.

## TUMOR DORMANCY AND RELAPSE

**Figure 1:**
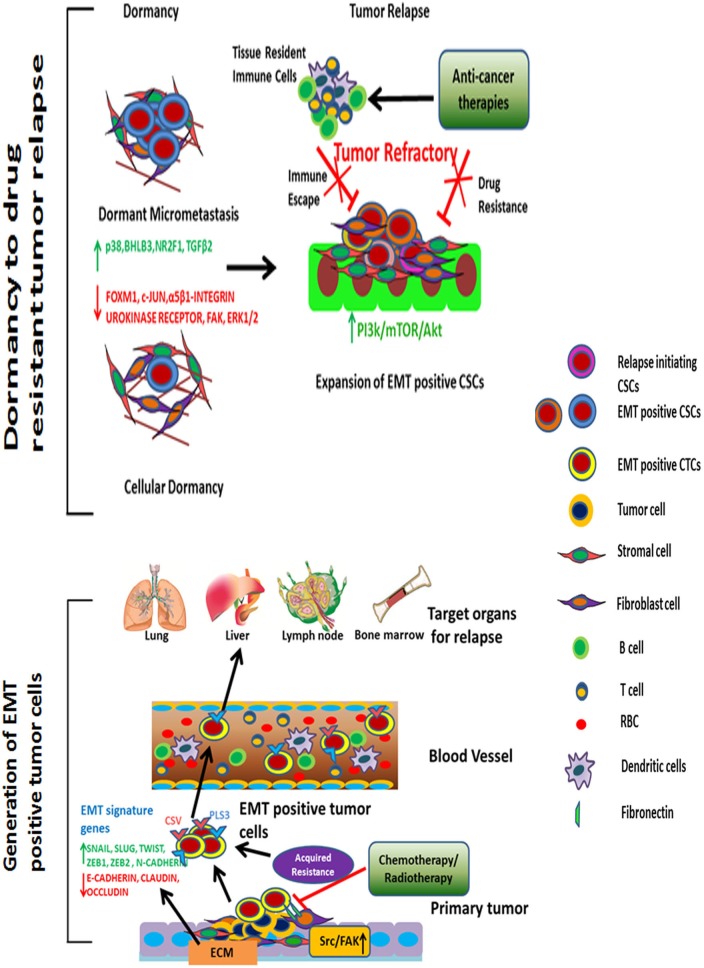
Tumor relapse driven by EMT-positive CTCs and CSCs Upon anti-cancer therapy of primary tumors, EMT-positive CTCs are detected in large numbers in the peripheral blood. These CTCs migrate through organs such as the liver, lungs, lymph nodes, and bone marrow. Once the tumor cells arrive at their site of relapse, they remain dormant for an extended period and transform into CSCs. ECM remodeling; p38α, NR2F1, and TGFβ2 signaling; and inhibition of ERK1/2, FAK, FOXM1, and c-JUN pathways facilitate dormancy. Furthermore, tumor-associated tissue environments provide an embedded niche to protect these cells from anti-cancer therapies or any other lethal damage. Under ambient conditions, with ECM remodeling and activation of proliferative, angiogenic signaling pathways, EMT-positive CSCs undergo proliferation to initiate recurrence. These cells are highly resistant to anti-cancer drugs and are capable of evading immune surveillance.

Tumor dormancy is a state of clinical remission in which cancer cells remain occult, i.e., indiscernible and asymptomatic for an extended period of time. Primary tumors often employ this strategy as a means of resisting the first line of treatment. The phenomenon of dormancy is associated with numerous epithelial tumors, including melanoma and breast, prostate, liver, and thyroid cancer with variable latency periods [24-29]. Dormancy is frequently observed in patients with cancer who have undergone frequent chemo- or radiation therapy [30, 31]. Thus, it is critical to delineate gene signatures associated with the sequential stages of dormancy including mitotic-arrest in dividing tumor cells, the angiogenic switch, and the escape from immune surveillance and transformation into relapse initiating cells (Figure 1).

In the era of translational studies and extensive genomic sequencing, numerous genes have been linked to dormancy in different types of cancer [32-35]. For example breast and prostate cancers, NR2F1, SHARP1, BMP7^high^and COCO^low^signatures induce quiescence and delayed metastasis [36]. Many investigations recently interpreted that EMT-transformed cells are linked with decreased proliferation or quiescence [37-39]. Notably, the EMT program drives tumor cells to become quiescent CTCs. Identifying the molecular characteristics of EMT positive CTCs and CSCs during the latency period are thus instrumental to determine whether these cells relapse or remain dormant.

According to experimental and clinical studies, the microenvironments of certain organs such as the bone marrow, lung, liver, and brain promote dormancy [40]. The host microenvironments in these tissues maintain reciprocal signaling with CTCs and CSCs and thus induce the expression of pro-dormancy genes. Furthermore these cancer cells are embedded in a niche that provides a shield from immune surveillance, extending the period of dormancy.

Survival signals rather than proliferative ones can be identified in dormant tumor cells and used to prevent recurrence. In multiple myeloma, bortezomib treatment causes tumor cells to enter a quiescent phase owing to activation of the unfolded protein response pathway [41]. Inhibiting eIF2α dephosphorylation in this type of cancer using the GADD34-PP1c inhibitor decreased the number of dormant tumor cells and reduced recurrences in this type of cancer [41]. In breast cancer, low expression of extracellular-signal-regulated kinase (i.e., ERK) and high levels of p38α were detected in quiescent cancer cells [42]. Activation of p38α induced at least three transcription factors- p53 (R213Q), BHLHB3 and NR2F1 and inhibited the expression of FOXM1 and c-JUN, which are associated with G1-S transition [42, 43]. Some of these dormancy signature genes were found to delay recurrence in breast and prostate cancers by suppressing malignant behavior [44]. Furthermore knockdown or systemic inhibition of p38α *in vivo*showed that dormant cells were capable of regaining tumorigenicity [45]. A recent study using a head and neck cancer model identified that transforming growth factor-β 2 (TGFβ2) was increased in dormant cells [45]. TGFβ2 created a unique signal through TGFβRIII to induce canonical pathway SMAD1/2/5 to upregulate p27 and induce non-canonical activation of p38 for dormancy [45].

In addition to p38α and TGFβ2, the composition of the ECM has the potential to determine proliferation and dormancy. Adhesion to the ECM initiates intracellular signaling pathways that can increase cell cycle progression, motility, survival, and other metastatic phenotypes of tumor cells. For example, downregulation of the urokinase receptor in squamous cell carcinoma (HEp3) inactivates α5β1-integrins [46]. Subsequently, focal adhesion kinase (FAK) signaling is inhibited owing to the cells inability to bind to fibronectin. This results in dormancy of cancer cells. Conversely, dormancy to proliferative response in a fibrotic environment requires collagen-I mediated integrin β1 signaling, which requires activation of Src and FAK to phosphorylate myosin light chain kinase in an ERK dependent manner [47]. Clearly cytoskeletal rearrangement and ECM composition are critical in determining whether tumor cells will remain dormant or metastasize. Thus, inhibiting the growth promoting changes in an ECM-associated microenvironment may help prevent relapse.

## EMT AND CANCER ADVANCEMENT

The EMT program is now known to facilitate the metastatic spread and progression of cancer cells from the site of the primary tumor to the surrounding tissues and distant organ(s). The identification and biological characterization of the EMT inducing transcription factors Snail, Slug and Twist showed the cascade of the tissue remodeling process of epithelial tumors [48, 49]. Overexpression of these EMT signatures changes the polarity of epithelial cells such that they acquire the morphological and biochemical traits of mesenchymal cells. Numerous genes linked with EMT, evidence that this program is essential for tumor cells to circumvent apoptosis, anoikis, oncogene addiction, and cellular senescence and to escape immune surveillance [50].

Understanding and targeting the adaptive growth of EMT driven cancer cells could lengthen progression-free survival [51]. A prospective study of 46 patients with liver cancer showed the EMT markers twist and Vimentin in 84.8% and 80.4% of those patients' CTC samples respectively [52]; tumor progression was closely correlated with the presence of EMT positive CTCs in those patients. In patients with non–small cell lung cancer (NSCLC), resistance to EGFR inhibitors was associated with EMT induction [53, 54]; in this subset, EMT may have been promoted through Zeb1 and Src activation upon overexpression of the growth factor CRIPTO1 [53]. A seminal study by Shao et al highlighted that a loss of K-Ras signaling was compensated by the transcriptional coactivator YAP1 to maintain the EMT program during relapse in a murine lung cancer model [55]. Similarly, the functional study with High Mobility Group A1 (HMGA1) protein has emphasized its role as a key regulator of the mesenchymal transition and linked with stem-like phenotypes in breast cancer [56]. Apart from dynamics of cellular proteins, metabolic reprogramming is an essential step to maintain EMT state for CSCs [57]. Under nutrient starvation condition, EMT positive CSCs utilizes glycolytic and ketogenic end products to catabolize exogenous mitochondrial fuel [57, 58]

These findings suggest that the differentiation state of tumor cells contributes significantly to acquired drug resistance. The mechanisms by which tumor cells sustain the EMT phenotype in the relapse state are highly diverse between different types of cancer. The enhancement of mesenchymal-like features may epigenetically reprogram epithelial cancer cells to adapt well to new microenvironments and thus may contribute to distant recurrence.

## CTCS AND RISK OF RELAPSE

CTCs have gained huge importance in the design of therapeutic regimens and monitoring cancer progression in the era of personalized medicine. Owing to advancements in single-cell molecular analysis, CTCs are considered a precursor for metastatic transformation and a predictive factor of tumor relapse. Compared with the traditional single-biopsy approach, the analysis of CTCs captures a broad range of genomic variations. With the use of next-generation sequencing, CTC profiles are a powerful clinical indicator for the transition from chemotherapeutic susceptibility to chemoresistance. Also, the genomic landscape obtained from these sequencing data greatly facilitates the identification of druggable therapeutic targets.

**Figure 2:**
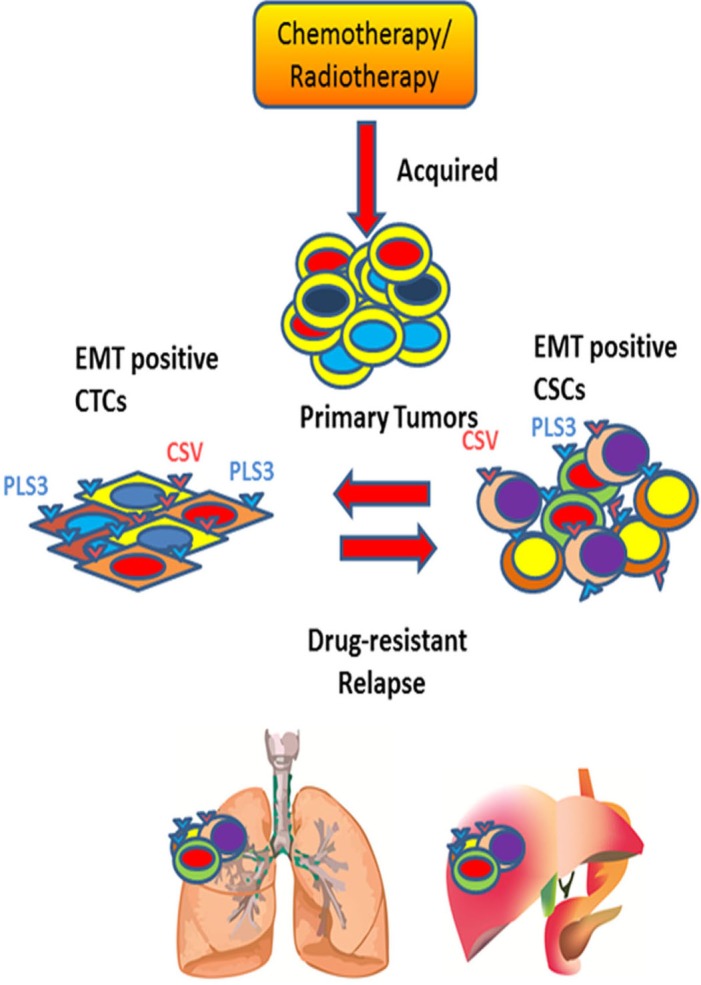
Understanding the dynamic equilibrium between EMT positive CTCs and CSCs to define tumor relapse Periodic chemo- or radiotherapy on primary tumor induces EMT positive tumor cells. These EMT positive tumor cells are transformed into quiescent CTCs upon entering into the bloodstream. These EMT positive CTCs express Plastin 3 (PLS3) and cell surface Vimentin (CSV) on its surface. During relapse phase, EMT positive CTCs reprogram into drug-resistant EMT positive CSCs under ambient condition to cause poor survival rate for cancer patients.

CTCs are heterogeneous and can be broadly classified into three categories-epithelial, transitioning from epithelial to mesenchymal and mesenchymal. We will focus on epithelial and EMT CTCs and their association with metastatic potential and acquired drug resistance in adult cancers. Epithelial-origin CTCs are detected in the peripheral circulation and are believed to shed periodically from primary or metastatic tumor sites (Figures 1, 2). Extensive, seminal studies in the past decade have implicated EMT and CSCs in metastasis and relapse [15, 18, 59].

Acquisition of the EMT phenotype in CTCs can indicate the risk of relapse and survival (Figure 1). Compared with epithelial CTCs detected by the conventional markers EpCAM and cytokeratin, the high rates of EMT-positive CTCs were associated with prognosis in patients with hepatocellular carcinoma [52]. In breast, prostate, liver, colorectal, head and neck, pancreatic, endometrial, and lung cancers, the number of CTCs exhibiting EMT markers increased from early- to late-stage cases [4, 16, 52, 60, 61]. In a comparative study between early and metastatic breast cancer cases, CTCs expressing the EMT markers vimentin and twist increased from ~73-77% to ~100% of CTCs [16]. In another study, 14 of 52 primary breast cancers between stages I and III received neoadjuvant therapy [10]. Interestingly, EMT-inducing transcription factors were overexpressed in neoadjuvant therapy-treated patients compared with those not treated with neoadjuvant therapy. Similarly, in patients with colorectal cancer, the novel marker Plastin3 identified the most aggressive CTCs undergoing EMT in one-third of 711 patients with colorectal cancers [11]; these Plastin3-positive CTCs showed inducible staining of the EMT marker vimentin.

Another novel marker, cell surface Vimentin (CSV), has recently been shown to detect EMT-positive CTCs in breast and colorectal cancer patients [62, 63]. In a pilot study of 58 patients with metastatic breast cancer, CSV antibodies demonstrated superior sensitivity (85% vs 48%) and specificity (94% vs 83%) compared with EpCAM-based detection for progressive disease upon treatment. This difference could be attributed to a shifting of the CTC population toward drug-resistant, dormant, or both phenotypes. Also, the low detection of CTCs by the EpCAM antibody was likely due to its nuclear localization upon disease progression. These studies clearly concluded the possibility of shortcomings in U.S. Food and Drug Administration–approved EpCAM-based CTC capture techniques [16, 64, 65].

More than 300 clinical trials are using CTC counts as an indicator for disease progression and overall survival (Table 2) [66, 67]. These clinical trials reflect the translational significance of CTC for monitoring therapeutic responses to adjuvant therapies. In general, higher numbers of CTCs (≥5 per 7.5 ml of blood) were associated with shorter median survival and higher tumor burden (Table 2). Upon chemotherapeutic treatment, a decrease in CTC count was associated with an improvement in median overall survival. Hence, CTC kinetics has the potential to indicate whether to maintain current medication or switch therapy. However, most of these clinical trials used EpCAM as a CTC marker, which may mean that the assessments of the pharmacodynamics of the drugs under trial are not optimally accurate. It is imperative to include the markers Plastin3 and CSV to detect EMT-positive CTCs for comprehensive, more precise characterization of CTCs.

**Table 2:** Prognostic significance of CTC counts in phase II and III clinical trials in cancers of various tissues

Tumor Tissue	CTC detection rate	Phase of Trial	Prognostic relevance	References
Breast	91% (n = 41 of 45)	II	42.9% of patients had 12 months of survival with CTCs (≥1).	[114]
Breast	11.2%(n = 51 of 455)	III	Not applicable.	[115]
Breast	39% (n = 148 of 378)	II	75% of trastuzumab-treated group showed CK19-negative CTCs.	[116]
Colon	37.5% (n = 180 of 480)	III	41% of CTC-positive (≥5) patients had 24 months survival.	[117]
Lung	44.4% (n = 8 of 18)	II	76.5% patients showed favorable CTC counts.	[118]
Lung	78% (n = 32 of 41)	II	18% of patients with ≥5 CTCs converted to favorable CTCs (<5).	[119]
Ovary	32.1% (n = 216 of 672)	III	CTC count was not correlated with survival.	[120]
Prostate	66% (n = 263 of 400)	III	Median progression-free survival times, 25.1 months (<3 CTCs) and 16.2 months (≥3 CTCs).	[121]
Prostate	71.5% (n = 88 of 123)	I/II	47% of patients with ≥2 positive and 28% of patients with <2 CTC biomarkers showed distant relapse.	[122]
Prostate	35.4% (n = 11 of 31)	II	Overall survival rate for 36 months was positive for 55% of patients with 1 positive and 42% of patients with 2-3 positive CTC biomarkers.	[123]
Pancreas	37.5% (n = 19 of 51)	II	Median overall survival times, 17.4 to 25.3 months (<2 CTCs) and 12.4 months (≥2 CTCs).	[124]
Skin	~13-17.5% (n = 44-56 of 320)	III	Unable to correlate with disease characteristics owing to low CTC counts.	[125]
Skin	86% (n = 214 of 269)	III	Increased progression-free survival with decreased CTC counts.	[126]

Characterizing the EMT phenotype in CTCs is not sufficient to explain their transformation to a proliferative state at distant organs; it is critical to shed light on the variable duration of dormancy and how these cells are breaking quiescence and are modified into relapse-initiating cells at a secondary site. Recent studies validated the existence of stem cell–like CTCs, which have the ability to self-renew, clonally expand, and initiate tumors, like CSCs can [20, 59, 68, 69]. In 2013, Sun et al reported the CSC biomarkers CD133 and ABCG2 in EpCAM-positive CTCs in 82 patients with hepatocellular carcinoma [70], and they identified the nuclear localization of β-catenin in 10 of 17 of these patients with EpCAM-positive CTCs. The authors concluded that EpCAM-positive CTCs with stem cell–like phenotypes might represent a subset of CTCs with a more aggressive phenotype, earlier recurrence, and worse survival. Further studies are required to explore these stem cell–like CTCs to predict the recurrence timeframe and determine the therapeutic window of treatment for better survival.

## CSCS AND RELAPSE

CSCs are a rare subset of tumor cells that bear properties of stem cells, and they show the greatest diversity in cancer progression. Recently, substantial progress has been made in understanding CSCs by characterizing genetic and epigenetic changes occur in their dormant and relapsed stages (Figures 1, 2); however, the surface markers may not unequivocally enrich all CSCs. To date, researchers have identified a few surface markers that enrich various CSCs from the primary tumor for the majority of cancer types. Tumor dormancy and therapeutic refractoriness in different types of cancer are due largely to CSCs and their clonal evolution [71-74]. However, because of the repeated refinement of the CSCs on the basis of new markers, it is difficult to categorize the exact or overlapping populations responsible for promoting the processes of dissemination, intravasation, dormancy, and relapse. Also, self-replicative and non-differentiating cancer stemloids are a topic of considerable interest to pursue effective anti-cancer therapy [74, 75]. These stem-like cells play a seminal role in therapy-resistant relapse due to diverse oncogenic mutations in their clonal populations [75]. Thus, selectively targeting cancer stemloids might provide better therapeutic response for cancer patients.

Our increasing understanding of the molecular biology and aberrantly activated cellular pathways of CSCs has revealed a number of novel targets for targeted therapeutic regimens that have successfully reduced CSCs both *in vitro*and in preclinical models (Table 3). An example is that upregulation of anti-apoptotic pathway has been detected to maintain mesenchymal state and chemoresistance in breast cancer cells [76]. Using this molecular concept in preclinical study showed that BH3-mimetics were capable to remove both epithelial and mesenchymal HMLE (Human Mammary with Large T and TERT) cells [76]. Therefore, drugs suppressing CSCs hold great promise for redefining cancer therapy in advanced-stage cases. However, CSCs undergo dynamic clonal modification during the metastatic cascade, chemotherapeutic treatments, dormancy, and relapse. Because of their highly heterogeneous nature, relapse-initiating CSCs must be captured and characterized, as most conventional anti-cancer therapies have limited success in eradicating them in patients. A recent study on prostate cancer cells showed that EMT-positive CSCs exhibit resistance to radiation therapy via the PI3K/Akt/mTOR pathway [19]. The biologic link between EMT phenotypes and CSCs has recently been evidenced by epigenetic programming in many types of cancer [77, 78]. In breast cancer cells, Snail interacts with methyltransferase G9a to recruit DNA methyltransferase at the E-cadherin promoter region to silence its expression under low-glucose conditions [78]. In malignant pediatric brain tumor ATRT (Atypical teratoid/rhabdoid tumor), activated STAT3 regulates EMT phenotypes in association with Snail in cisplatin resistant cells [79]. A recent work found that nuclear localized PKCθ acts as a chromatin-anchored switch for EMT to induce expression of mesenchymal genes [78]. Furthermore, the long noncoding RNA Hotair is overexpressed upon TGFβ pathway activation in many cases of cancer [80]; Hotair interacts with polycomb repressive complex 2 to promote methylation at the promoter regions of epithelial genes and involved EMT progression [80, 81].

**Table 3:** Novel therapeutic compounds targeting CSCs in various tissue-specific cancers ATRA, all-trans retinoic acid; AML, acute myelogenous leukemia; CML, chronic myelognous leukemia; MM, multiple myeloma.

Tissue stem cells	Drug	*In vivo study*	*In vitro* inhibition	References
Breast	ATRA	No	Mammosphere	[127]
Breast	IMD-0354+ Doxorubicin	Yes	Sphere	[128]
Breast	Salinomycin	Yes	Mammosphere	[129]
Brain	Disulfiram	No	Ubiquitin-proteasomal pathway	[130]
Brain	γ-secretase inhibitor	Yes	Notch pathway	[131]
Blood (AML)	ABT-263	Yes	Oxidative phosphorylation	[132]
Blood (CML)	FTY720	Yes	PP2A agonist	[133]
Blood (MM)	Palcitaxel-Fe_3_O_4_	Yes	Not tested	[134]
Colon	Metformin + FuOx	No	Colonosphere	[135]
Colon	CC188	No	Carbohydrate epitope on the surface	[136]
Colon	α-DLL4	Yes	Not tested	[137]
Gallbladder	Emodin	Yes	ABCG2 pump	[138]
Liver	Lupeol	Yes	Hepatosphere	[139]
Lung, Breast and Ovary	VS-5584	No	PI3K-mTOR	[140]
Pancreas	GDC-0449	No	Hedgehog pathway	[141]
Pancreas	GNT-61	Yes	Hedgehog pathway	[142]

It has been demonstrated that a subset of isolated CSCs expresses EMT phenotypes in numerous cancers [82-84]. Unfortunately, no suitable marker can enrich EMT-transformed CSCs that are phenotypically different from primary tumor–derived CSCs. To avoid relapse, these cells must be detected in a dormant phase, which may last from a few months to many years.

Some studies validated stem cell–like properties in CTCs [20, 59], suggesting that CSCs transformed into these CTCs and then became dormant. During relapse, they may then become EMT-positive CSCs that proliferate as relapse-initiating cells forming aggressive tumors. Considering the immense clinical significance of these CSCs, it is important to develop strategies to enrich these cells for molecular understanding of relapse. Application of conventional surface markers of CSCs has proven difficult owing to the dynamic clonal evolution of these cells in response to chemotherapy, dormancy, and new tumor microenvironments.

One potential alternative strategy for enriching CSCs is using CTC markers, which can enrich EMT-positive populations, as EMT-positive cancer cells are indicators of aggressive relapse in cancer patients [85]. Thus, isolating EMT-transformed CSCs using CTC surface markers from the relapse site and from the primary tumor could provide a comprehensive picture of the etiologies of relapse. Substantial and systematic research focusing on drug-resistant, relapse-initiating CSCs could promote the development of effective treatment for aggressive cancer, and the identification and culture of these CSCs could be a powerful tool in the investigation of cancer relapse. Also, the identification of aberrant pathways in relapse-initiating CSCs could facilitate the development of therapies for patients for whom traditional chemotherapies and radiation treatments have poor clinical outcomes.

## SUMMARY

The etiology of tumor recurrence with a variable time frame remains elusive. Major obstacles include the heterogeneity of tumors in patients and the difficulty of capturing residual drug-resistant tumor cells. Thus, recent research has aimed to identify suitable clinical models that can accurately catalog the steps of cancer recurrence. Clinical studies indicate that harnessing EMT-transformed CTCs and CSCs could shed light on the transition from dormancy to relapse in cancer patients. Future therapeutic studies of CSCs should focus on EMT positive CSCs or relapse-initiating tumor cells rather than just CSCs enriched from primary tumors. The molecular and cellular plasticity of EMT-positive cells needs to be characterized to categorize aberrant molecular pathways and heterotypic interactions with tumor microenvironments. Furthermore, ECM remodeling that supports the EMT program in tumor cells to initiate drug resistance and relapse is required to allow more in-depth tracing.

Currently, neoadjuvant therapy is recommended for patients who are at risk of recurrence after resection of the primary tumor. However, because of the dynamic interaction between tumor microenvironments and cancer cells, EMT-positive CSCs frequently undergo genetic drift and clonal evolution, so novel pharmacologic agents that demonstrate better therapeutic efficacy than current neoadjuvant therapies need to be generated. As translational research is streamlined toward more personalized therapy, suppressing EMT-transformed CTCs and CSCs should prove useful for preventing relapse and extending the lifespans of patients with recurrent cancer.

## Abbreviations

CTCs: Circulating tumor cells
CSCs: Cancer stem cells
EMT: Epithelial mesenchymal transition
NAT: Neo adjuvant therapy
NR2F1: Nuclear receptor subfamily group 2
TGFβ2: Transforming growth factor β 2
FAK: Focal adhesion kinase
ERK: Extra-cellular-signal related kinase
MLCK: Myosin light chain kinase
BHLHB3: Basic helix-loop-helix domain containing, class B, 3
PLS3: Plastin 3
